# *Porphyromonas gingivalis* Stimulates TLR2-PI3K Signaling to Escape Immune Clearance and Induce Bone Resorption Independently of MyD88

**DOI:** 10.3389/fcimb.2017.00359

**Published:** 2017-08-08

**Authors:** Hasnaa Makkawi, Shifra Hoch, Elia Burns, Kavita Hosur, George Hajishengallis, Carsten J. Kirschning, Gabriel Nussbaum

**Affiliations:** ^1^Institute of Dental Sciences, Hebrew University-Hadassah Faculty of Dental Medicine Jerusalem, Israel; ^2^Department of Microbiology, School of Dental Medicine, University of Pennsylvania Philadelphia, PA, United States; ^3^Institute of Medical Microbiology, University of Duisburg-Essen Essen, Germany

**Keywords:** *P*. *gingivalis*, immune evasion, TLR2 signaling, MyD88, PI3 kinase, neutrophils, macrophages

## Abstract

*Porphyromonas gingivalis* is a gram-negative anaerobic periodontal pathogen that persists in dysbiotic mixed-species biofilms alongside a dense inflammatory infiltrate of neutrophils and other leukocytes in the subgingival areas of the periodontium. Toll-like receptor 2 (TLR2) mediates the inflammatory response to *P. gingivalis* and TLR2-deficient mice resist alveolar bone resorption following oral challenge with this organism. Although, MyD88 is an adaptor protein considered necessary for TLR2-induced inflammation, we now report for the first time that oral challenge with *P. gingivalis* leads to alveolar bone resorption in the absence of MyD88. Indeed, in contrast to prototypical TLR2 agonists, such as the lipopeptide Pam3CSK4 that activates TLR2 in a strictly MyD88-dependent manner, *P. gingivalis* strikingly induced TLR2 signaling in neutrophils and macrophages regardless of the presence or absence of MyD88. Moreover, genetic or antibody-mediated inactivation of TLR2 completely reduced cytokine production in *P. gingivalis*-stimulated neutrophils or macrophages, suggesting that TLR2 plays a non-redundant role in the host response to *P. gingivalis*. In the absence of MyD88, inflammatory TLR2 signaling in *P. gingivalis*-stimulated neutrophils or macrophages depended upon PI3K. Intriguingly, TLR2-PI3K signaling was also critical to *P. gingivalis* evasion of killing by macrophages, since their ability to phagocytose this pathogen was reduced in a TLR2 and PI3K-dependent manner. Moreover, within those cells that did phagocytose bacteria, TLR2-PI3K signaling blocked phago-lysosomal maturation, thereby revealing a novel mechanism whereby *P. gingivalis* can enhance its intracellular survival. Therefore, *P. gingivalis* uncouples inflammation from bactericidal activity by substituting TLR2-PI3K in place of TLR2-MyD88 signaling. These findings further support the role of *P. gingivalis* as a keystone pathogen, which manipulates the host inflammatory response in a way that promotes bone loss but not bacterial clearance. Modulation of these host response factors may lead to novel therapeutic approaches to improve outcomes in disease conditions associated with *P. gingivalis*.

## Introduction

Bone resorption is a prominent feature of chronic conditions of microbial etiology, such as osteomyelitis and periodontitis. In periodontitis, polymicrobial dysbiotic communities induce inflammation that leads to erosion of the soft and hard tissues that support the teeth, and eventually to tooth loss (Armitage, 1996; Pihlstrom et al., 2005). *Porphyromonas gingivalis*, a Gram-negative asaccharolytic anaerobic oral bacterium, was shown to remodel a symbiotic community into a dysbiotic one, thereby driving the inflammatory process and leading to osteoclast activation and bone resorption in the oral cavity (Holt and Bramanti, 1991; Socransky and Haffajee, 1992; Hajishengallis et al., 2011; Barth et al., 2012). In chronic periodontitis, bacteria persist within an environment rich in activated neutrophils and monocyte/macrophages, inflammatory cytokines, enzymes, and reactive oxygen species (Delima and Van Dyke, 2003; Ryder, 2010; Nussbaum and Shapira, 2011). In fact, inflammation provides the nutritional support for the asaccharolytic communities of bacteria such as *P. gingivalis* by releasing tissue breakdown products such as peptides and heme-containing compounds (Hajishengallis, 2014). Considering inflammation as a nutrient source can explain the clinical observation that bacterial numbers increase in accordance with the degree of host inflammation. However, activated immune cells kill bacteria by intracellular and extracellular mechanisms, so to thrive in a highly inflamed milieu, *P. gingivalis* must dissociate the beneficial effects of inflammation from bactericidal activity (Nussbaum and Shapira, 2011; Hajishengallis et al., 2012; Hajishengallis, 2014). The role of the innate immune receptor Toll-Like Receptor 2 (TLR2) in the host response to *P. gingivalis* exemplifies this dissociation—sensing of infection and the inflammatory response are driven by TLR2, however, the response does not lead to killing of *P. gingivalis*. Indeed, the absence of TLR2, rather than its presence, confers resistance to disease (Burns et al., 2006), although the underlying mechanisms are incompletely understood. TLR2-driven inflammation is normally linked to bactericidal activity and to osteoclast activation and bone loss through signaling initiated by the intracellular adaptor protein MyD88 (Pandey et al., 2014). Therefore, to evade bactericidal activity, *P. gingivalis* disrupts MyD88 activity, as demonstrated in neutrophils (Maekawa et al., 2014) and *in vivo* (Mizraji et al., 2017), but surprisingly it does not disrupt TLR2-driven inflammation (Burns et al., 2010). Although, MyD88 has been linked to a number of pathological inflammatory conditions (Kfoury et al., 2014; Kassem et al., 2015a), we demonstrate here that *P. gingivalis* induces bone resorption independently of MyD88. Moreover, the MyD88-independent TLR2 activation induced by *P. gingivalis* stimulates PI3K signaling that drives inflammation but at the same time depresses phagocytosis and enables phagocytosed bacteria to escape lysosomal degradation. Therefore, activation of TLR2-PI3K occurs independently of MyD88 and is critical for *P. gingivalis* to escape immunity and drive inflammatory bone resorption. Understanding the mechanisms that uncouple inflammation from bactericidal activity can lead to novel therapeutic approaches for chronic inflammatory tissue damage associated with dysbiotic microbiota.

## Materials and methods

### Reagents

LY 294002 and LY 303511 were from Sigma-Aldrich (Rehovot, Israel). Pam3CSK4 was obtained from InvivoGen (San Diego, CA). T2.5 monoclonal antibody (mAb) against mouse and human TLR2 was from Hycult Biotech (Uden, Netherlands), mAb 1A6 was a gift from Greg Elson (NovImmune, Geneva, Switzerland), and isotype control mAbs were from BioLegend (San Diego, USA). Recombinant proteins were from Peprotech (Rehovot, Israel). LysoTracker® Red DND-99 was from ThermoFischer Scientific (MA, USA).

### Bacterial growth

*P. gingivalis* (ATCC strains 381 and 53,977 were used in this study) was cultured for 48 h in Wilkins broth (Oxoid, Hampshire, England) without additional nutrients, under anaerobic conditions in Oxoid™ AnaeroJar™ 2.5 L at 37°C. An OD value of 0.1 (650 nm) was determined to correlate to 10^10^ CFU per ml.

### Mice

C57BL/6 were from Envigo (Rehovot, Israel), and *Tlr2*^−/−^, and *Myd88*^−/−^ mice backcrossed to the C57BL/6 background were a kind gift from Dr. S. Akira (Osaka University, Osaka, Japan). All mice were housed at the SPF unit of Hebrew University. *Tlr2*^−/−^*/Myd88*^−/−^ double-knockout mice were generated by screening F2 progeny of *Tlr2*^−/−^ females crossed with *Myd88*^−/−^ males as described previously (Burns et al., 2010). The institutional animal care and use committee of the Hebrew University of Jerusalem approved all experiments and experiments complied with the guidelines of the National Research Council Guide for the Care and Use of Laboratory Animals (NIH Publication no. 85-23, revised 1996).

### Cell lines

RAW 264.7 and THP1 cell lines were obtained from the American Type Culture Collection (ATCC, USA). RAW264.7 cells were maintained in DMEM and THP-1 cells in RPMI (Sigma-Aldrich, Israel) and both were supplemented with 10% fetal calf serum, 2 mM L-glutamine, penicillin (100 units/ml), streptomycin (100 μg/ml; Biological Industries, Israel). In addition, 1% 1M HEPES, and sodium pyruvate were added to the culture medium of THP-1 cells. The cell lines were cultured at 37°C and 5% CO2. THP-1 cells were differentiated to mature macrophages by the addition of PMA each for 72 h.

### Bone marrow isolation and differentiation

Mice were sacrificed and the tibia and femur were extracted and cleaned from excess flesh. The ends of each tibia and femur were clipped with dissecting scissors and the bone marrow cells were flushed with ice cold PBS^−/−^ using a syringe with 27-G needle. The pooled bone marrow was then separated by gentle pipetting, followed by filtration through a sterile 70-μm nylon cell strainer to remove cell clumps and bone particles. The filtrated cells were centrifuged for 5 min at 1,500 rpm and resuspended in complete DMEM. To prepare Bone Marrow Macrophages (BMM), cells were plated 5 × 10^6^ cells/ plate and cultured in complete DMEM supplemented with 15% L cell supernatant as a source of M-CSF, and 15% horse serum. Media was replaced after 3 days and cells were collected for use after 1 week. To stimulate, BMM were plated in triplicate at 400,000 cells/well, in 96-well flat bottom plates in complete DMEM. Bone marrow neutrophils were prepared by negative or positive selection from pooled bone marrow cells. Negative selection was performed using the EASYSEP® magnet cell selection system (STEMCELL Technologies, Vancouver, Canada) according to the manufacturer's instructions. Positive selection was performed using the MACS® mouse Anti-Ly-6G MicroBead Kit (Miltenyi Biotec Inc., CA, USA) according to manufacturer's instructions.

### Peritoneal exudate cells (PECs)

Mice were administered 1 ml Glycogen (Sigma) and after 4 h the mice were sacrificed and PECs were collected from the peritoneal cavity by washing with cold ${\text{Ca}}_{2}^{+}$/${\text{Mg}}_{2}^{+}$ free PBS.

### *P. gingivalis* oral infection

Alveolar bone loss was induced by *P. gingivalis* oral infection as previously described (Baker et al., 2000). Briefly, mice (*n* ≥ 8) were treated with Sulfamethoxazole (0.4% solution in drinking water) for 10 days, followed by 3-days without antibiotics. Mice were infected with live *P. gingivalis* in PBS (4 × 10^9^ CFU) containing 2% carboxymethylcellulose (“vehicle”) using a round-tipped feeding needle three times at 2-day intervals. Control groups were treated with vehicle alone. In experiments for locating *P. gingivalis* in host tissues, 1, 7, and 14 days after the last challenge mice were sacrificed and gingiva and lung were obtained. Tissue samples were immediately frozen in −20°C until they were processed for DNA isolation. In experiments measuring alveolar bone resorption 6 weeks after the first challenge mice were sacrificed and maxillae were scanned by μCT (SCANCO Medical, Switzerland). Three-dimensional alveolar bone loss was quantified as reported (Wilensky et al., 2005; Steinmetz et al., 2016; Mizraji et al., 2017). Approximately 180 slices 12 μm wide were scanned for each sample, covering the entire bucco-palatal aspect.

### Detection of *P. gingivalis* in murine tissues

Tissue samples were incubated in lysis buffer (200 mM NaCl, 5 mM EDTA, 0.2% SDS, 100 mM Tris pH 8, 100 μm/ml Proteinase K) overnight at 55°C. After centrifugation and transfer of clear supernatant, DNA was precipitated by adding isopropanol. DNA was washed, dried, and resuspended in double-distilled water. *P. gingivalis* was identified in host tissue by using specific primers recognizing a 432 bp fragment of the *P. gingivalis* 16S gene. Forward primer sequence 5′-AGAGTTTGATCCTGCTCAG-3′ and reverse primer sequence 5′-CAATACTCGTATCGCCCGTTATTC -3′. Reaction conditions used were: 94°C for 2 min followed by 40 cycles of 30 s at 94°C, 30 s at 63°C, 1 min at 72°C. PCR products were analyzed on 1% agarose gel with 100 bp DNA ladder (NEB, USA).

### Cytokine analysis

Cytokine levels were determined by ELISA using mouse and human Elisa MAX TM sets (Biolegend, San Diego, CA), according to the manufacturer's instructions.

### Phagocytosis assay

Macrophages were seeded in 96 well flat bottom plates and challenged with FITC-labeled *P. gingivalis*. To label the bacteria, *P. gingivalis* was incubated with 0.1 mg/ml FITC (Sigma, Israel) in carbonate buffer (pH 9.5) for 20 min at RT and then extensively washed in PBS. Following challenge, macrophages were washed twice with PBS and then incubated with 1 ml of 1X trypan blue for 1 min to quench extracellular fluorescence from bacteria attached to the external surface of the macrophages. Cells were washed twice with PBS^−/−^ and fluorescence was determined using a GENios Microplate Reader (Tecan, Männedorf, Switzerland).

### Intracellular survival assay

Intracellular survival was determined using an antibiotic protection assay. 10^6^ macrophages/well were plated in 6-well plates and challenged with live *P. gingivalis* at multiplicity of infection (MOI) of 10 for 1 h. Cells were then washed twice and treated with metronidazole and gentamycin for an additional hour to kill all remaining extracellular bacteria. Following antibiotic treatment, cells were washed twice and incubated in medium without antibiotics for an additional hour. Cells were then lysed by incubation in ice cold DDW for 20 min. Serial dilutions of lysates were plated on blood agar plates (Novamed, Jerusalem, Israel), and incubated under anaerobic conditions for 7 days to determine the number of Colony Forming Units (CFU).

### Phago-lysosomal maturation

Thirty thousand cells/well were plated in U-Slide 8 well-IBIDI tissue culture treated chamber slides (Ibidi, Martinsried, Germany). Cells were blocked as indicated for 1 h prior to challenge with FITC-labeled *P. gingivalis* for another hour. Lysotracker red (50 mM) was added to cells 10 min prior to fixing. Cells were washed twice, fixed with 2% formaldehyde for 10 min and then washed with PBS for 5 min and IBIDI mounting medium was applied (Ibidi). Multi-projection images were obtained using a NIKON confocal fluorescent microscope at 60X magnification.

### Statistical analysis

The 2-Tailed *t-*test was used for statistical evaluation of all the results. Values are shown for data that reached a significance of *P* ≤ 0.05 (^*^), *P* ≤ 0.01 (^**^), *P* ≤ 0.005 (^***^). Bars show mean and standard deviation (*s.d*.; Prism v.5, GraphPad Software Inc. San Diego, USA).

## Results

### Oral infection with *P. gingivalis* induces alveolar bone resorption in the absence of MyD88

**Figure 1:**
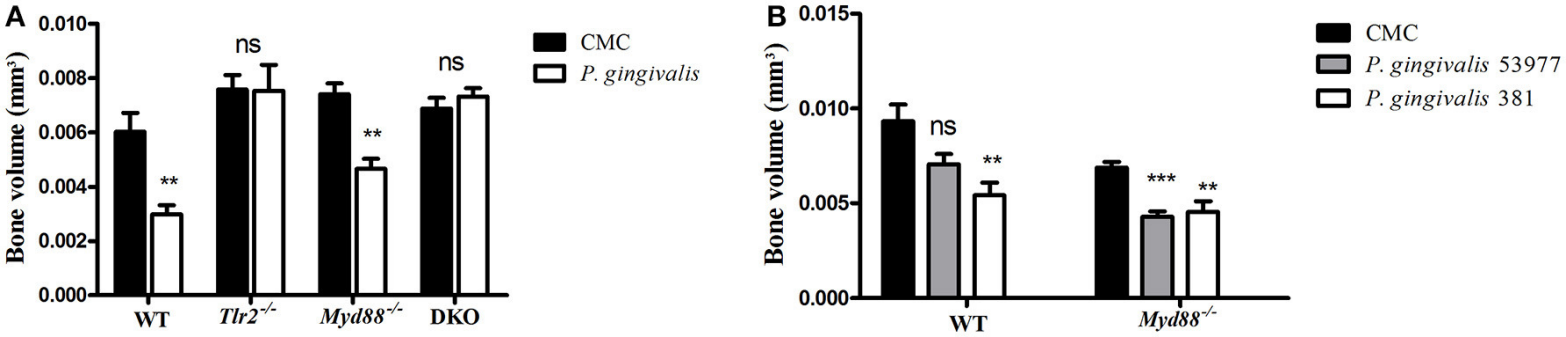
Experimental periodontitis induced by oral challenge with *P. gingivalis*. Groups of mice were administered *P. gingivalis* in CMC vs. CMC alone by repeated oral gavage. Six weeks later, the maxillae were harvested and alveolar bone volume was measured by μCT from the cemento-enamel junction to a reference line. The residual bone volume of *P. gingivalis* infected mice was compared to vehicle-treated mice (*n* = 8–10 per group). **(A)** WT, *Tlr2*^−/−^, *Myd88*^−/−^, and *Tlr2/Myd88* double knock-out mice (DKO) infected with *P. gingivalis* ATCC 381. **(B)** An independent experiment examined bone loss in WT vs. *Myd88*^−/−^ mice infected with *P. gingivalis* ATCC 381 vs. *P. gingivalis* ATCC 53977. Ns, non-significant. **P ≤ 0.01, ***P ≤ 0.005.

Repeated oral challenge of mice with *P. gingivalis* leads to its colonization followed by chronic inflammation, osteoclast activation, and resorption of alveolar bone surrounding the teeth (Baker et al., 2000). We and others demonstrated that TLR2-deficient mice are resistant to alveolar bone loss following oral challenge with *P. gingivalis* (Burns et al., 2006; Papadopoulos et al., 2013), consistent with the role of TLR2, together with C5aR, in mediating the inflammatory response to *P. gingivalis* that leads to osteoclast differentiation (Maekawa et al., 2014). Since the canonical signaling pathway of TLR2 requires the adaptor protein MyD88, we set out to test if the phenotype of resistance to alveolar bone resorption would be recapitulated in MyD88-deficient mice. Surprisingly, in contrast to TLR2-deficient mice, the absence of MyD88 did not prevent *P. gingivalis* from inducing alveolar bone resorption, as shown for a non-encapsulated *P. gingivalis* strain (ATCC 381), and an additional strain that expresses a polysaccharide capsule (ATCC 53977; Figures 1A,B). We next tracked the clearance of *P. gingivalis* by PCR from mouse tissues 24 h and 7 days following the last oral challenge. Consistent with the importance of TLR2 in bacterial immune evasion, *P. gingivalis* was not detectable in the gingival tissue of most *Tlr2*^−/−^ mice at 24 h following the third oral challenge, and was not detected in any mice 7 days later (Table 1). In contrast, *P. gingivalis* was detected in all WT and MyD88-deficient mice 24 h following the third challenge, and was still detectable in the gingiva of most mice 7 days later. We also tested for *P. gingivalis* in the lungs of orally-infected mice as a measure of bacterial dissemination (either via aspiration during oral challenge or through spreading infection). Whereas, *P. gingivalis* was detected in the lungs of all WT and *Myd88*^−/−^ mice 24 h after the last challenge, there was no evidence of bacteria in the lungs of *Tlr2*^−/−^ mice. These data collectively suggested that MyD88 does not mediate the TLR2-dependent evasive effects that promote *P. gingivalis* persistence, required for disease. Accordingly, to examine whether *P. gingivalis* infection leads to TLR2-dependent but MyD88-independent bone resorption, we infected TLR2/MyD88 double knock-out mice. First, we observed that *P. gingivalis* could be detected in the double knock-out mice where its survival was similar to the single MyD88 knock-out mice (Table 1), indicating that the absence of TLR2 (and hence the ability of *P. gingivalis* to evade immunity) is without serious consequences when MyD88 is also absent (hence MyD88 is required for immune clearance). Strikingly, despite the fact that the double knock-out mice were colonized by *P. gingivalis*, they were completely resistant to infection-driven bone resorption (Figure 1A), suggesting an absolute requirement for TLR2 in pathologic bone loss in this model. Taken together, these results demonstrate that MyD88-independent TLR2 signaling is necessary for bacterial persistence, and that TLR2-driven inflammation promotes oral infection-driven bone resorption even in the absence of the canonical TLR2 adaptor protein MyD88.

**Table 1:** Detection of *P. gingivalis* in mouse tissue following the third oral challenge.

	**Gingiva (%)**	**Lung (%)**	**Gingiva (%)**	**Lung (%)**
	**24 h**		**7 days 4**	
WT	100	100	50	0
*Myd88^−/−^*	100	100	75	0
*Myd88^−/−^*/*Tlr2^−/−^*	100	100	66	0
*Tlr2^−/−^*	40	0	0	0

### Neutrophils and macrophages produce TNF in response to *P. gingivalis* through a MyD88-independent, PI3K-dependent pathway *in vitro*

**Figure 2:**
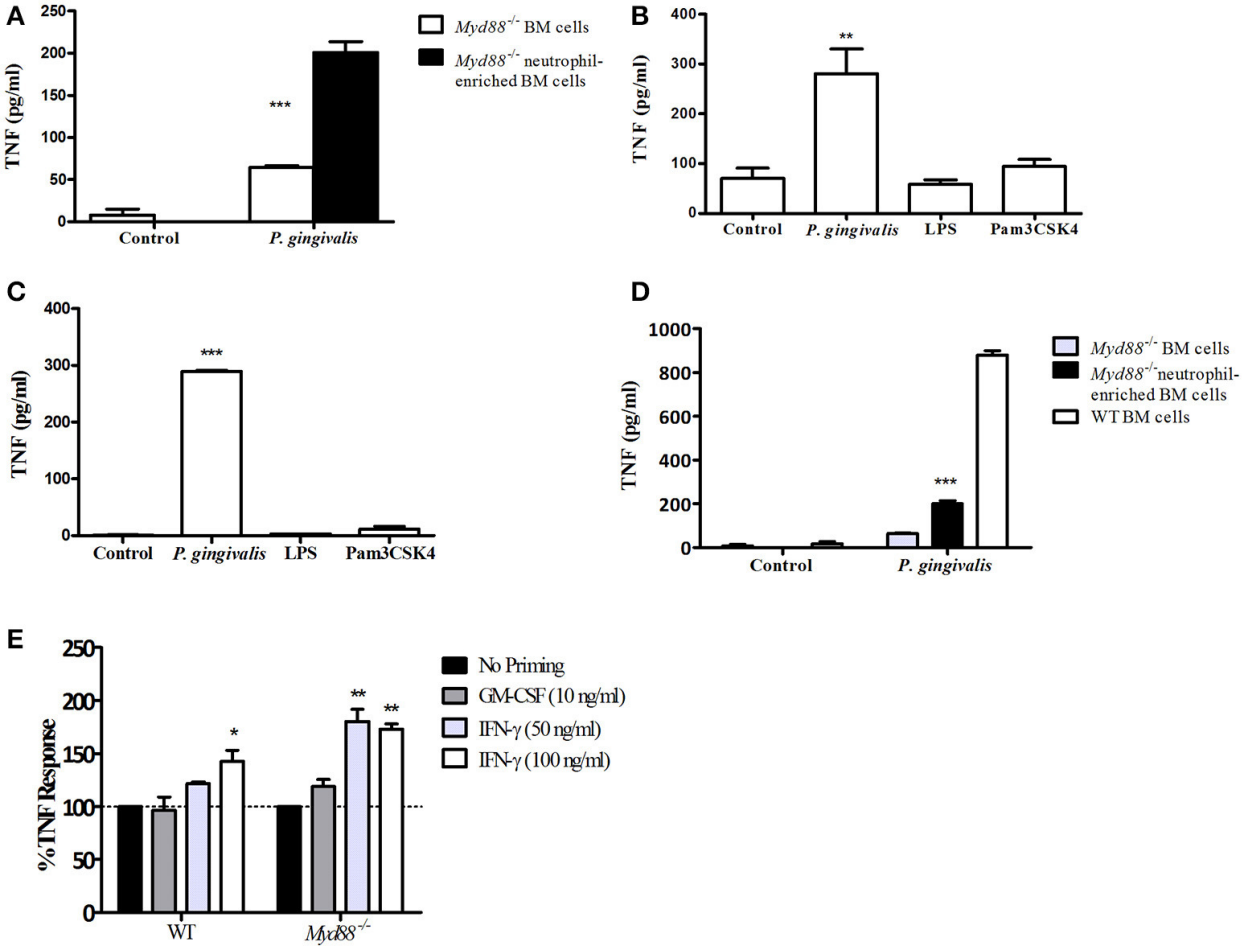
*Myd88*^−/−^ neutrophils respond to challenge with *P. gingivalis*. **(A)** Total *Myd88*^−/−^ bone marrow cells and neutrophil-enriched bone marrow cells were plated 4^*^10^5^ cells/well in a 96-well plate and incubated with *P. gingivalis* (MOI 100). **(B)** Glycogen-induced *Myd88*^−/−^ PECs, or naïve bone marrow *Myd88*^−/−^ Ly6G+ neutrophils isolated by positive selection **(C)** produce TNF-α in response to *P. gingivalis* challenge but not in response to *S. minnesota* LPS (1 μg/ml) or Pam3CSK4 (10 μg/ml), or buffer control. **(D)** The response of *Myd88*^−/−^ total and neutrophil enriched BM was compared to the response of WT BM cells. **(E)** BM cells were collected from *WT and Myd88*^−/−^ mice and primed with GM-CSF or IFN-γ for 2 h prior to challenge with *P. gingivalis* **(E)**. The percent increase in TNF produced in response to *P. gingivalis* challenge in primed cells vs. unprimed cells is shown. **(A–E)** Supernatants were collected after overnight challenge and the level of TNF in the supernatants was measured by ELISA. One representative experiment is shown in each case. Experiments were repeated 3–5 times. ^*^P ≤ 0.05, ^**^P ≤ 0.01, ^***^P ≤ 0.005.

To dissect the mechanism by which *P. gingivalis* can induce a TLR2-dependent but MyD88-independent inflammatory response, we next studied the role of MyD88 in response to infection with *P. gingivalis in vitro*, and compared this response to that induced by the prototypical lipopeptide Pam3CSK4, which stimulates TLR2 in a strictly MyD88-dependent manner. We previously reported that *in-vivo*, the cytokine response to subcutaneous challenge with *P. gingivalis* is similar in WT and *Myd88*^−/−^ mice (Burns et al., 2010). Since >70% of the cells responding to subcutaneous challenge are neutrophils (Steinmetz et al., 2016), we hypothesized that TLR2-dependent, MyD88-independent signaling may be most prominent in this cell type. *In vitro* activation of a mixed population of bone marrow (BM) cells from MyD88-deficient mice with *P. gingivalis* induced significant cytokine production (Figure 2A). Consistently, moreover, enrichment of the neutrophil population by negative selection (achieving roughly 60% Ly6G^+^ cells) further enhanced the response (Figure 2A). *Myd88*^−/−^ peritoneal exudate cells (PECs) collected 4 h following glycogen administration (~89% neutrophils), and positively-selected Ly6G^+^ bone marrow neutrophils (>96% purity) also responded to *P. gingivalis* challenge *in vitro*, but notably failed to respond to either Pam3CSK4 or *E. coli* LPS (Figures 2B,C). However, WT BM cells secreted more TNF when stimulated with *P. gingivalis* than *Myd88*^−/−^ BM cells (Figure 2D), suggesting that both MyD88-dependent and independent pathways are activated in response to challenge *in vitro*.

We next hypothesized that factors known to prime cells responding to infection, such as IFN-γ and Granulocyte-Macrophage Colony-Stimulating Factor (GM-CSF), may contribute to the ability of *P. gingivalis* to induce TLR2-dependent, MyD88-independent inflammatory signaling. To test this notion, WT and *Myd88*^−/−^ BM neutrophils were exposed to GM-CSF or IFN-γ for 2 h prior to challenge with live *P. gingivalis*. Priming cells with either compound alone did not produce significant amounts of TNF. Although GM-CSF priming had no effect on the *Myd88*^−/−^ neutrophil response to *P. gingivalis*, IFN-γ priming significantly increased the response to bacterial challenge (Figure 2E). In contrast, IFN-γ priming did not induce MyD88-independent TNF production in response to Pam3CSK4 (data not shown). Thus, IFN-γ enhances TLR2, MyD88-independent signaling in neutrophils in response to *P. gingivalis* but not necessarily to other TLR2 agonists.

**Figure 3:**
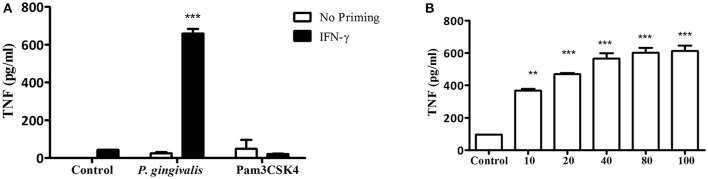
IFN-γ priming enables TLR2-dependent, MYD88-independent signaling in macrophages in response to *P. gingivalis* challenge. **(A)** Naïve *Myd88*^−/−^ BMM vs. BMM primed with IFN-γ (100 ng/ml) for 2 h were challenged with *P. gingivalis* (MOI 100) vs. Pam3CSK4 (10 μg/ml). In **(B)** *Myd88*^−/−^ BMM primed with IFN-γ were challenged with increasing MOI of *P. gingivalis*. **(A,B)** Supernatants were collected after overnight stimulation and tested for TNF by ELISA. ^**^P ≤ 0.01, ^***^P ≤ 0.005.

**Figure 4:**
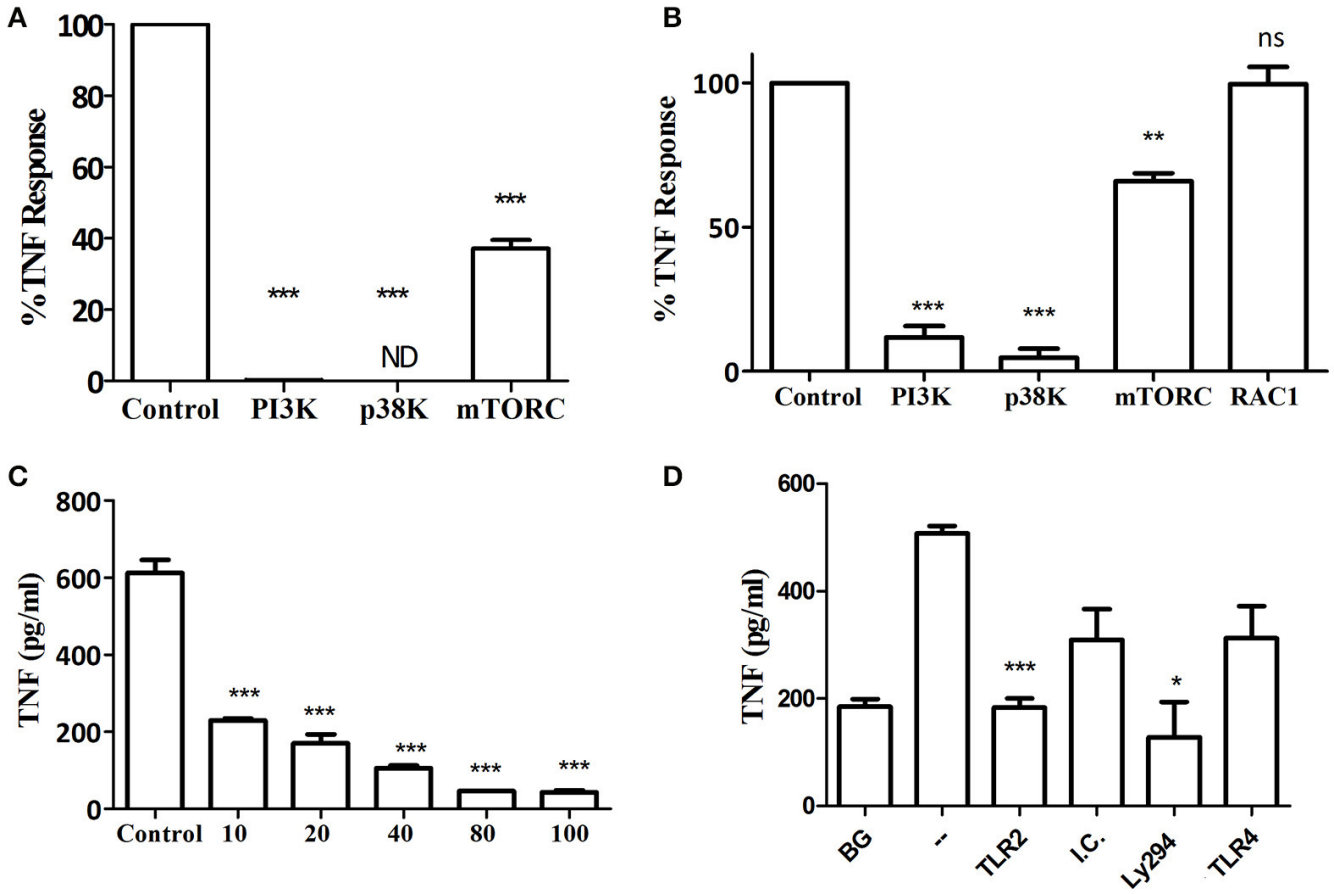
Kinase involvement in the *Myd88*^−/−^ response to *P. gingivalis*. **(A)** Ly6C+ BM neutrophils or **(B–D)** BMM were prepared from *Myd88*^−/−^ mice and primed with IFN-γ (100 ng/ml for 2 h). **(A,B)** Inhibitors for PI3K (LY 2940002 100 μM), p38 MAPK (SB 202190 50 μM), mTORC1 (RaPamycin 60 nM) and RAC1 inhibitor (NSC 23766, 50 μM) were added 30 min before challenge with *P. gingivalis* (MOI 100). DMSO was used as a control at the highest concentration used in the inhibitor wells. Supernatants were collected after overnight stimulation and the percent inhibition of TNF production is shown. **(C)** *Myd88*^−/−^ BMM were similarly primed and Ly294 was added at increasing concentrations 30 min prior to challenge with *P. gingivalis*. **(D)** *Myd88*^−/−^ BMM were primed with IFN-γ and antibodies (20 μg/ml anti-TLR2 or TLR4 vs. isotype control, I.C.) or LY294 were added prior to challenge with *P. gingivalis*. ^*^P ≤ 0.05, ^**^P ≤ 0.01, ^***^P ≤ 0.005.

In addition to neutrophils, macrophages predominate in the host response to subcutaneous or oral infection with *P. gingivalis*, where they regulate the neutrophil response (Lam et al., 2014; Steinmetz et al., 2016). Interestingly, *Myd88*^−/−^ BM-derived macrophages (BMM) required IFNγ priming in order to respond to increasing MOI of *P. gingivalis* infection (Figures 3A,B). Consistent with the *Myd88*^−/−^ neutrophil response, priming did not enable *Myd88*^−/−^ BMM to respond to Pam3CSK4 (Figure 3A). We previously showed that *P. gingivalis* promotes its survival *in vivo* by cross-talk signaling between TLR2 and C5aR1 in a manner dependent on phosphatidylinositol-3-OH kinase (PI3K; Maekawa et al., 2014). We now show that inhibition of PI3K completely abrogated MyD88-independent cytokine production in response to *P. gingivalis in vitro* (Figures 4A–C), suggesting that PI3K is a crucial component of the MyD88-independent TLR2 pathway. Inhibition of p38K, a downstream MAPK, also blocked cytokine production in *Myd88*^−/−^ cells (Figures 4A,B), whereas blocking other factors implicated in TLR2 signaling such as mTORC1 (Lorne et al., 2009) or RAC1 (Arbibe et al., 2000; Harokopakis et al., 2006) either partially disrupted (mTORC1) or did not affect (RAC1) MyD88-independent signaling (Figures 4A,B). We further confirmed that MyD88-independent signaling in response to *P. gingivalis* is dependent on TLR2 using specific blocking antibodies. Indeed, blocking TLR2 or inhibition of PI3K on *Myd88*^−/−^ BMM reduced the *P. gingivalis*-induced response in a dose-dependent manner (Figure 4C), and to background levels, whereas blocking TLR4 had no effect (Figure 4D). Therefore, IFNγ priming facilitates a distinctive TLR2-PI3K signaling pathway that is completely independent of MyD88, leading to inflammatory cytokine production in response to *P. gingivalis*.

### TLR2-PI3K signaling is non-redundant in the murine and human macrophage cytokine response to *P. gingivalis*

**Figure 5:**
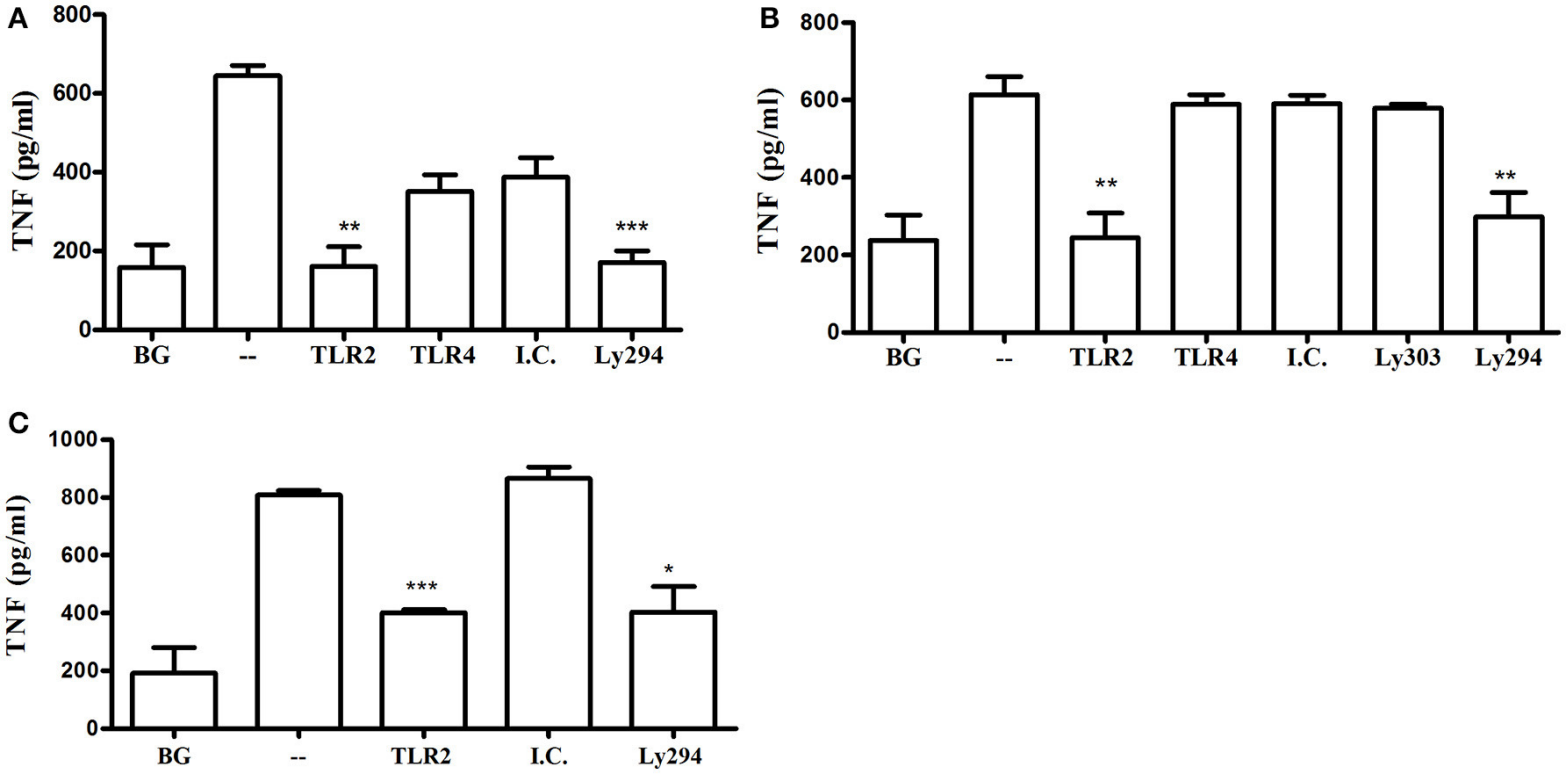
TLR2-PI3K plays a non-redundant role in the murine and human macrophage response to *P. gingivalis*. WT murine BMM **(A)**, RAW264.7 macrophages **(B)**, and PMA-differentiated human THP-1 cells **(C)** were primed with IFN-γ. TLR2 and TLR4 were inhibited with blocking antibodies vs. isotype control (I.C.) for 1 h and PI3K was blocked with LY294 prior to challenge with *P. gingivalis* (MOI 10). Supernatants were collected after overnight incubation and TNF was measured by ELISA. Background (BG) represents IFN-γ primed cells not challenged with *P. gingivalis*. Cells challenged with *P. gingivalis* without any blocker are referred to in the graphs as (–). Representative graphs of >3 repeats are shown. ^*^P ≤ 0.05, ^**^P ≤ 0.01, ^***^P ≤ 0.005.

Previous studies using macrophages from gene knockout mice suggested that MyD88-dependent TLRs, such as TLR4 (Papadopoulos et al., 2013) or TLR9 (Kim et al., 2015), contribute to cytokine production in response to *P. gingivalis*. In line with these findings, even though MyD88-deficient cells responded to *P. gingivalis* in the present study, the response is diminished compared to WT cells. These studies suggest that cells can respond to *P. gingivalis* through multiple redundant pathways. We therefore next determined the importance of TLR2-PI3K signaling in response to *P. gingivalis* using WT mouse and human macrophages. Blocking TLR2, but not TLR4, and inhibition of PI3K, reduced cytokine production to near background levels in primary BMM and the murine RAW264.7 macrophage cell line responding to infection with *P. gingivalis* (Figures 5A,B). The specificity of the blocking antibodies to TLR2 and TLR4 was confirmed using murine and human macrophages challenged with Pam3CSK4 (for the inhibitory antibody to TLR2) and LPS (for the antibody to TLR4). As expected, inhibition of PI3K did not affect cytokine production induced by Pam3CSK4 (data not shown). The role of TLR2 and PI3K in the response to *P. gingivalis* was also confirmed in the human THP-1 macrophage cell line primed with IFNγ (Figure 5C). Therefore, in WT cells with functional TLRs and MyD88, the TLR2-PI3K pathway induced by *P. gingivalis* plays a non-redundant role in cytokine production.

### TLR2-PI3K signaling induced by *P. gingivalis* downregulates phagocytosis and enhances intracellular survival by blocking phago-lysosomal maturation

**Figure 6:**
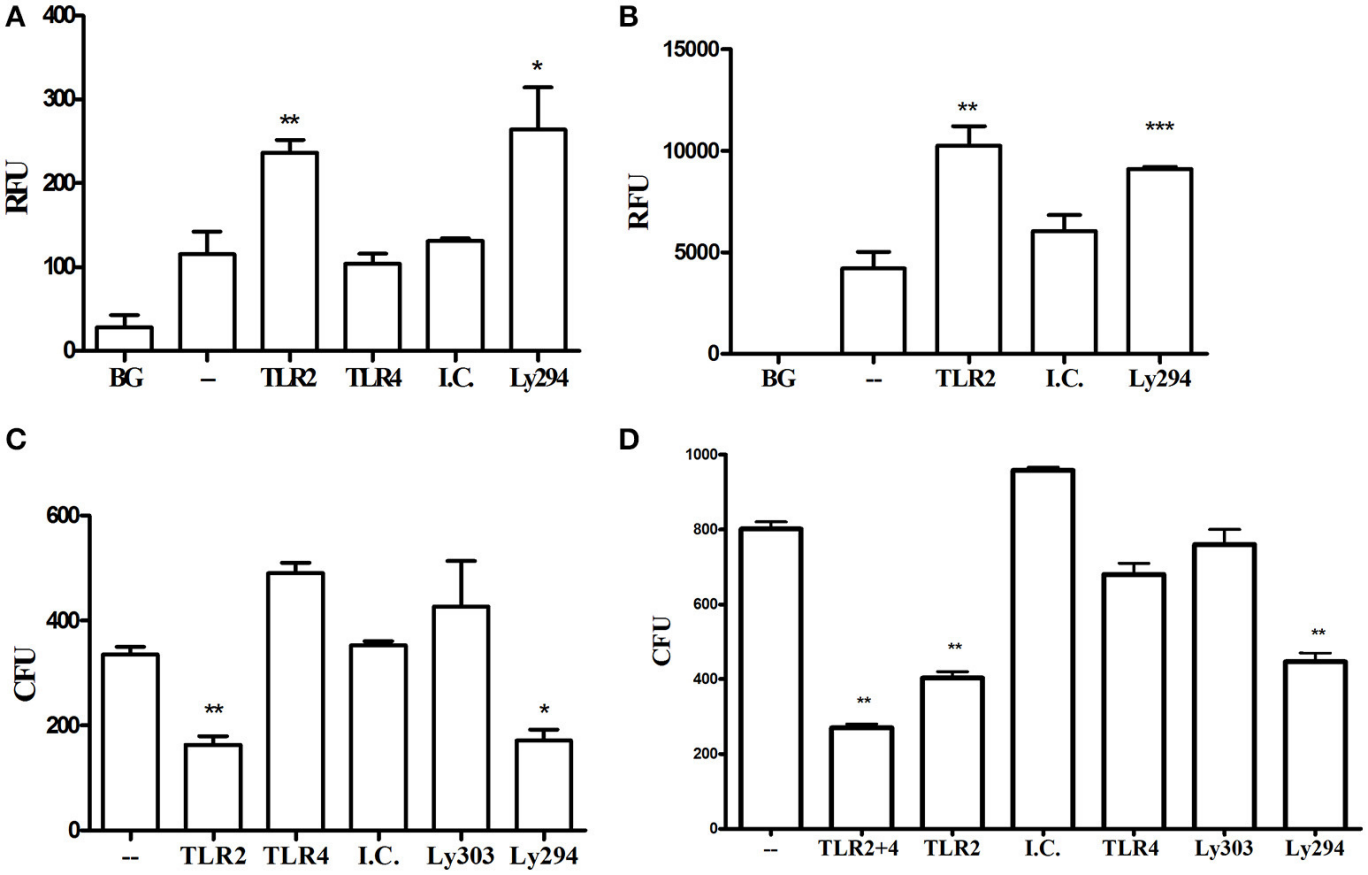
TLR2-PI3K signaling suppresses phagocytosis and enhances intracellular survival. **(A)** RAW264.7 or **(B)** PMA-differentiated THP-1 cells were treated with blocking antibodies or PI3K inhibitor and then challenged with FITC-labeled *P. gingivalis* at MOI 10 for 1 h. Cells were then washed, extracellular fluorescence was quenched with trypan blue, and phagocytosis was determined using a fluorescence plate reader (RFU, relative fluorescence units). **(C,D)** RAW 264.7 cells were treated with TLR blocking antibodies or the PI3K inhibitor prior to challenge with *P. gingivalis* at MOI 10 for 1 h. Cells were then washed and extracellular bacteria were killed by incubating the cells with Metronidazole and Gentamycin for 1 h. Cells were allowed to recover in fresh media for an additional hour after which they were lysed by DDW for 20 min and lysates were plated on blood agar plates in serial dilution. CFU were enumerated after 7 days of anaerobic growth. ^*^P ≤ 0.05, ^**^P ≤ 0.01, ^***^P ≤ 0.005.

*In vivo*, TLR2 signaling promotes the survival of *P. gingivalis* and enables infection to drive dysbiosis and alveolar bone resorption (Maekawa et al., 2014). The results above demonstrate that the TLR2-PI3K pathway drives inflammation in response to *P. gingivalis*, but the inflammatory response is disconnected from immune bacterial clearance *in vivo*. To identify the mechanism underlying the TLR2-dependent evasion of *P. gingivalis* clearance, we next determined the effects of TLR2-PI3K signaling *in vitro* on the ability of macrophages to phagocytose and eradicate *P. gingivalis*. Phagocytosis was measured in a florescence plate-based assay by labeling the bacteria with FITC and quenching extracellular florescence with Trypan Blue after phagocytosis. In stark contrast to results with other organisms whose phagocytosis is promoted by TLR2 and/or PI3K signaling (Shin et al., 2009; Giraldo et al., 2010; Fang et al., 2014), blocking TLR2 or PI3K, but not TLR4, significantly enhanced WT murine and human macrophage phagocytosis of *P. gingivalis* (Figures 6A,B). Next, we tracked the fate of phagocytosed bacteria using an antibiotic protection assay (Hajishengallis et al., 2017). Despite reduced entry of *P. gingivalis* into the macrophages when the TLR2-PI3K pathway is active, we observed higher intracellular bacterial burden under this condition than when the TLR2-PI3K pathway was blocked (Figure 6), suggesting that the *P. gingivalis*-induced TLR2-PI3K pathway suppresses phagocytosis but, even if phagocytosed, the bacteria have a way to escape killing. Indeed, blocking either TLR2 or PI3K significantly improved eradication of phagocytosed bacteria, whereas blocking TLR4 had no effect (Figure 6C). TLR4 does not mediate the bactericidal activity observed when TLR2 is inhibited since co-inhibition of TLR2 and TLR4 led to similar diminished *P. gingivalis* survival as when TLR2 is blocked alone (Figure 6D). Thus, the TLR2-PI3K pathway is responsible for both inflammatory signaling and bacterial evasion from killing via a two-pronged mechanism; (i) reduced phagocytosis and (ii) enhanced intracellular survival within those cells that do phagocytose bacteria.

**Figure 7:**
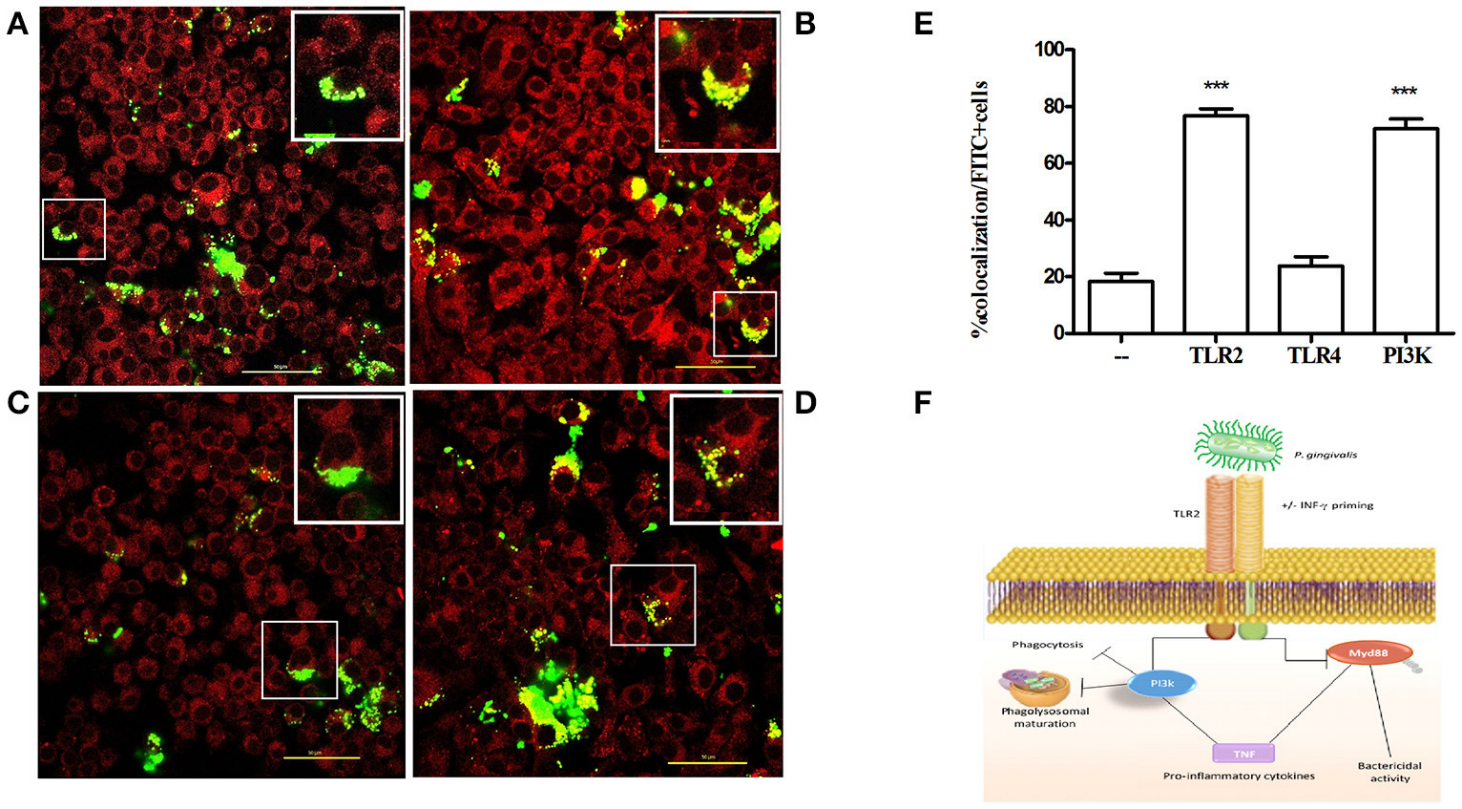
TLR2-PI3K signaling enhances intracellular survival by blocking phago-lysosomal maturation. RAW 264.7 cells were seeded at 3 × 104 cells/ well in Ibidi 8 well m-slides. The cells were untreated **(A)** or treated with anti-TLR2 **(B)**, anti-TLR4 **(C)**, or with the PI3K inhibitor LY 294002 **(D)** for an hour. Cells were then infected with FITC-labeled *P. gingivalis* at MOI 10 for 1 h. LysoTracker red was added at 50 nM for the last 10 min of infection. Cells were washed and fixed with 2% formaldehyde and mounted with mounting media. Images were captured using a NIKON confocal microscope at 60X magnification. Yellow color indicates co-localization of *P. gingivalis* (green) with lysosomes (red). In each field **(A–D)** the cell in the box is further magnified and shown in the upper right corner. **(E)** The percent of co-localization was determined by counting cells that demonstrate co-localization as a percentage of all FITC positive cells. **(F)** Schematic representation of the pathway used by *P. gingivalis* to evade bactericidal activity without preventing inflammation. ^***^P ≤ 0.005.

To investigate the mechanism by which the TLR2-PI3K pathway promotes the survival of internalized *P. gingivalis* bacteria, we tracked the intracellular fate of *P. gingivalis* in the presence or absence of TLR2-PI3K signaling. To this end, we labeled bacteria with FITC and labeled acidified vacuoles with the lysosomal marker lyso-tracker red, a dye that fluoresces in a low pH environment. Phagosome maturation and lysosomal fusion would lead to co-localization of the FITC signal with the LysoTracker signal. Cells were treated with inhibitors prior to challenge with FITC-labeled *P. gingivalis*, and Lyso-tracker was added 15 min prior to fixing the cells. When TLR2 or PI3K were inhibited, there were more internalized bacteria, consistent with the enhanced phagocytosis shown in Figure 6. However, in stark contrast to untreated or isotype control treated cells, when TLR2 or PI3K were inhibited most internalized bacteria were found in lysosomes (Figure 7). Therefore, TLR2-PI3K signaling enhances *P. gingivalis* intracellular survival by inhibiting phago-lysosomal maturation.

## Discussion

Repeated oral inoculation of mice with *P. gingivalis* leads to its colonization, quantitative and compositional alterations to the periodontal microbiota and the induction of an acute inflammatory infiltrate in the gingival tissue dominated by neutrophils and monocyte/macrophages, which in turn contribute to bone resorption surrounding the teeth (Baker et al., 2000; Burns et al., 2006; Hajishengallis et al., 2011; Papadopoulos et al., 2013). Our study shows for the first time that *P. gingivalis*-induced bone loss is TLR2-dependent but MyD88-independent. Our findings stand in contrast to the dominant role of MyD88 in TLR2-driven responses in general (Pandey et al., 2014), and in particular in the setting of osteoclastogenesis. MyD88 is essential for osteoclastogenesis induced by IL-1, and ligands of TLR4 or TLR2 (Sato et al., 2004; Kim et al., 2013). TLR2-dependent osteoclast differentiation and bone resorption in response to infection with *S. aureus* and *B. abortus* were also shown to be MyD88 dependent (Delpino et al., 2012; Kim et al., 2013). Finally, MyD88 is essential for TLR2-driven osteoclastogenesis *in vitro* in response to *P. gingivalis* or its isolated LPS (Zhang et al., 2011; Kassem et al., 2015b), and for alveolar bone loss induced by LPS from other oral pathogens (Madeira et al., 2013). Although, MyD88 plays a role in *in vitro* osteoclastogenesis involving direct interactions of *P. gingivalis* with osteoclast precursors (Zhang et al., 2011), the significance of MyD88 may be different in biologically more relevant settings that include all cellular players (i.e., both immune and bone cells). Indeed, the ability of live *P. gingivalis* to manipulate MyD88 in immune cells and induce TLR2 signaling via alternative adaptors results in the induction of TLR2-dependent, MyD88-independent inflammation that leads to bone loss. We earlier showed that MyD88 promotes immune clearance of *P. gingivalis* (Burns et al., 2010), which is consistent with our subsequent findings that the same pathogen induces MyD88 degradation in neutrophils (Maekawa et al., 2014) and in the oral tissue (Mizraji et al., 2017) to suppress the bactericidal activity mediated by MyD88. In the absence of both TLR2 and MyD88, *P. gingivalis* persists in the oral cavity but is unable to induce bone loss, further confirming the essential role of TLR2-dependent, MyD88-independent inflammation in bone loss that develops in response to infection.

Although *P. gingivalis* induced *Myd88*^−/−^ neutrophils to produce TNF *in vitro*, the cells remained unresponsive to challenge with the triacylated lipopeptide Pam3CSK4. Furthermore, exposure to IFNγ enhanced the neutrophil cytokine response to *P. gingivalis* without enabling the cells to respond to Pam3CSK4. Interestingly, *Myd88*^−/−^ macrophages required priming with IFNγ to respond to challenge with *P. gingivalis*, but still did not respond to Pam3CSK4. These findings suggest that the repertoire of available signaling molecules downstream to TLR2 is determined by the nature of the ligand and the type of the responding cell. *P. gingivalis* induces TLR2 crosstalk with complement and chemokine receptors (e.g., C5aR and CXCR4), expanding potential signaling pathways (Hajishengallis et al., 2008; Maekawa et al., 2014). Since they are the most abundant cell type responding to infection, neutrophils may be the prime target of immune evasion by *P. gingivalis*. To avoid killing, *P. gingivalis* degrades neutrophil MyD88 (Maekawa et al., 2014); nevertheless, we show that even unprimed neutrophils maintain inflammatory signaling in the absence of MyD88. Thus, inflammation, which benefits *P. gingivalis* by providing a nutrient source, is uncoupled from bactericidal activity mediated by MyD88 (Figure 7F).

As shown here, PI3K plays a central role in the MyD88-independent TLR2 response of neutrophils and macrophages challenged with *P. gingivalis*. PI3K may be recruited to TLR2 by the adaptor protein Mal/TIRAP (MyD88-adapter-like/TIR-domain-containing adaptor protein; Maekawa et al., 2014). Mal/TIRAP bridges between TLR2 and MyD88 (Pandey et al., 2014). However, since Mal/TIRAP can also interact with PI3K (Strassheim et al., 2004; Santos-Sierra et al., 2009), it may serve to bridge between TLR2 and PI3K in the absence of MyD88 (Maekawa et al., 2014).

The ability of *P. gingivalis* to exploit TLR2 signaling to block phagosome maturation and lysosomal fusion, thereby escaping intracellular killing in macrophages, constitutes a hitherto undescribed mechanism that can readily explain earlier observations. For instance, *P. gingivalis* was shown to persist at higher intracellular viable counts in WT than in *Tlr2*^−/−^ macrophages, although the mechanistic basis for this effect was uncertain (Hajishengallis et al., 2007). Moreover, disruption of membrane lipid rafts by cholesterol depletion was shown to promote the colocalization of *P. gingivalis* with lysosomes in macrophages (Wang and Hajishengallis, 2008). Although, the same study showed that lipid raft disruption impairs TLR2 signaling, the latter was not linked to alterations in phagolysosomal fusion. Our findings are consistent with TLR2-mediated escape of certain mycobacterial species from phagosomal degradation in monocytes (Weiss et al., 2008), although TLR2 engagement by other species promotes their phagosomal degradation (Gomes et al., 2008). Similarly, distinct PI3K classes modulate various steps of phagosome maturation, although in most cases PI3K activation promotes, rather than inhibits, maturation, and fusion to lysosomes (Thi and Reiner, 2012). Therefore, when PI3K acts to inhibit phagolysosomal fusion, this likely implies proactive microbial manipulation of host signaling, as shown in the present study. Identification of the particular PI3K classes induced by *P. gingivalis* downstream of TLR2 may shed light on the mechanism through which bacteria prevent lysosomal degradation.

In addition to its well-established pro-inflammatory properties, MyD88 signaling is critical to phagocytosis and phagosome maturation in response to gram-negative and gram-positive bacteria, as shown by numerous reports (Blander and Medzhitov, 2004; Yates and Russell, 2005). Therefore, besides serving as an alternative pathway for induction of inflammation, TLR2-PI3K signaling enables bacteria to escape phagocytosis and, even when phagocytosed, to escape lysosomal degradation. These results of the present study are consistent with, and explain mechanistically the reduced phagocytosis and enhanced *P. gingivalis* survival in *Myd88*^−/−^ mice (as compared to WT mice) in the subcutaneous chamber infection model where neutrophils and macrophages constitute the vast majority of cells responding to challenge (Burns et al., 2010; Steinmetz et al., 2016). Since neutrophils and monocyte/macrophages predominate in the oral niche where *P. gingivalis* thrives, it makes sense that *P. gingivalis* evolved to escape killing by these “professional killers.” However, TLR2 may play an opposite role in dendritic cells where TLR2 signaling was shown to enhance bactericidal activity (El-Awady et al., 2015; Hajishengallis et al., 2017). The cell-type specific factors that enable *P. gingivalis* to hijack TLR2 signaling in macrophages and neutrophils, but not in dendritic cells, have yet to be identified. *P. gingivalis* also invades and survives within epithelial and endothelial cells via manipulation of vesicle trafficking pathways (Dorn et al., 2001; Takeuchi et al., 2011) or through survival mechanisms within phagolysosomes (Yamatake et al., 2007). Although, the role of TLR2 in directing these events has not been fully explored, *P. gingivalis* stimulates and exploits PI3K signaling in gingival epithelial cells to promote its intracellular persistence (Yilmaz et al., 2004), suggesting that a similar evasion pathway exists in these diverse cell types.

*P. gingivalis* uncouples TLR2-driven inflammation from bactericidal activity by substituting PI3K in place of MyD88 signaling. Beyond the advantage to *P. gingivalis* itself, the creation of an inflammatory environment with impaired immunity benefits other inflammophilic anaerobic bacteria, further supporting the role of *P. gingivalis* as a keystone pathogen (Hajishengallis et al., 2012). Targeting the TLR2-PI3K escape pathway may improve therapeutic outcomes in *P. gingivalis*-driven periodontal bone loss.

## Author contributions

GN, GH, and CK conceived and designed experiments. HM, SH, EB, performed and analyzed *in vivo* and *in vitro* experiments. HM and GN wrote the first draft of the manuscript. GN, GH, KH edited text and figures.

### Conflict of interest statement

The authors declare that the research was conducted in the absence of any commercial or financial relationships that could be construed as a potential conflict of interest. The reviewer JP and handling Editor declared their shared affiliation, and the handling Editor states that the process nevertheless met the standards of a fair and objective review.
